# Nervous system diseases are associated with the severity and mortality of patients with COVID-19: a systematic review and meta-analysis

**DOI:** 10.1017/S0950268821000376

**Published:** 2021-02-15

**Authors:** Ya Gao, Yamin Chen, Ming Liu, Mingming Niu, Ziwei Song, Meili Yan, Jinhui Tian

**Affiliations:** 1 Evidence-Based Medicine Center, School of Basic Medical Sciences, Lanzhou University, Lanzhou 730000, China; 2 Evidence-Based Nursing Center, School of Nursing, Lanzhou University, Lanzhou 730000, China; 3 Key Laboratory of Evidence-based Medicine and Knowledge Translation of Gansu Province, Lanzhou University, Lanzhou 730000, China

**Keywords:** Cerebrovascular disease, COVID-19, meta-analysis, mortality, nervous system disease, severe illness

## Abstract

Coronavirus disease 2019 (COVID-19) has become a global pandemic. Previous studies showed that comorbidities in patients with COVID-19 are risk factors for adverse outcomes. This study aimed to clarify the association between nervous system diseases and severity or mortality in patients with COVID-19. We performed a systematic literature search of four electronic databases and included studies reporting the prevalence of nervous system diseases in COVID-19 patients with severe and non-severe disease or among survivors and non-survivors. The included studies were pooled into a meta-analysis to calculate the odds ratio (OR) with 95% confidence intervals (95%CI). We included 69 studies involving 17 879 patients. The nervous system diseases were associated with COVID-19 severity (OR = 3.19, 95%CI: 2.37 to 4.30, *P* < 0.001) and mortality (OR = 3.75, 95%CI: 2.68 to 5.25, *P* < 0.001). Specifically, compared with the patients without cerebrovascular disease, patients with cerebrovascular disease infected with COVID-19 had a higher risk of severity (OR = 3.10, 95%CI: 2.21 to 4.36, *P* < 0.001) and mortality (OR = 3.45, 95% CI: 2.46 to 4.84, *P* < 0.001). Stroke was associated with severe COVID-19 disease (OR = 1.95, 95%CI: 1.11 to 3.42, *P* = 0.020). No significant differences were found for the prevalence of epilepsy (OR = 1.00, 95%CI: 0.42 to 2.35, *P* = 0.994) and dementia (OR = 2.39, 95%CI: 0.55 to 10.48, *P* = 0.247) between non-severe and severe COVID-19 patients. There was no significant association between stroke (OR = 1.79, 95%CI: 0.76 to 4.23, *P* = 0.185), epilepsy (OR = 2.08, 95%CI: 0.08 to 50.91, *P* = 0.654) and COVID-19 mortality. In conclusion, nervous system diseases and cerebrovascular disease were associated with severity and mortality of patients with COVID-19. There might be confounding factors that influence the relationship between nervous system diseases and COVID-19 severity as well as mortality.

## Introduction

Coronavirus disease 2019 (COVID-19) is an infectious disease caused by severe acute respiratory syndrome coronavirus 2 (SARS-CoV-2) [1]. The World Health Organization (WHO) claims that COVID-19 has become a global pandemic on 11 March 2020 [2]. As of 30 October 2020, a total of 44 888 869 confirmed cases were reported globally, of which 1 178 475 cases had resulted in mortality [3].

The previous study showed that comorbidities in patients with COVID-19 are risk factors for adverse outcomes and cerebrovascular disease was associated with severe COVID-19 disease, which needs to be monitored in the intensive care unit (ICU) care [4]. A meta-analysis [5] suggested that cerebrovascular disease was associated with the increased poor composite outcome (RR = 2.04, 95%CI: 1.43 to 2.91, *P* < 0.001) and another meta-analysis [6] showed similar results. However, the existing meta-analyses only incorporated a small number of samples and most of the studies synthesised came from China. To date, there is still limited evidence regarding the concomitant association between nervous system diseases and COVID-19. Therefore, to address this gap in the literature, it is necessary to conduct a comprehensive meta-analysis. The purpose of this study was to clarify the association between nervous system diseases and severity or mortality in patients with COVID-19.

## Methods

To ensure the high quality of our work, we followed the Preferred Reporting Items for Systematic Reviews and Meta-Analyses (PRISMA) statement to conduct our study [7]. We registered this review protocol in the International Prospective Register of Systematic Reviews (PROSPERO, CRD42020180567).

### Eligibility criteria

We included case−control studies and cohort studies that met the following criteria: (1) patients were diagnosed with COVID-19 by a laboratory test or according to the World Health Organization interim guidance [8]; (2) reported data of pre-existing nervous system diseases, such as cerebrovascular disease, stroke and epilepsy between patients with severe and non-severe illness or between non-survivors and survivors; (3) published in English and Chinese.

We excluded studies with the following characteristics: (1) studies with a sample size of fewer than 20 patients; (2) studies did not report data related to nervous system diseases (e.g. cerebrovascular disease, stroke); (3) studies focused on only suspected cases or confirmed cases and suspected cases; (4) without comparisons (e.g. non-survivors *vs*. survivors); (5) review articles, protocols, guidelines, consensus, comments, abstracts, letters and editorials.

### Literature search

We comprehensively identified all potentially relevant articles through a systematic literature search of the electronic databases: PubMed, EMBASE.com, Web of Science and the Cochrane Central Register of Controlled Trials (CENTRAL). The searches were first performed on 8 May 2020 and updated on 10 October 2020. According to the indices of various databases, we used search terms as follows: ‘COVID-19’, ‘coronavirus disease-19’, ‘new coronavirus’, ‘2019-nCoV’, ‘novel corona virus’, ‘novel coronavirus’, ‘nCoV-2019’, ‘novel coronavirus pneumonia’, ‘2019 novel coronavirus’, ‘coronavirus disease 2019’, ‘SARS-CoV-2’, ‘severe acute respiratory syndrome coronavirus 2’, ‘clinical characteristic’, ‘clinical feature’, ‘risk factor’, ‘prognosis’, ‘comorbidit*’, ‘cerebrovascular disease*’, ‘nervous system disease*’, ‘brain’， ‘neurologic*’, ‘stroke’, ‘cerebral infarction’, ‘dementia’ and ‘epilepsy’. The search strategy of PubMed is shown in Appendix Word 1. We manually searched the reference lists of each included paper to identify potentially eligible studies.

### Study selection process

Records were managed by EndNote X8 (Thomson Reuters (Scientific) LLC Philadelphia, PA, US) software to exclude duplicates. At first, two authors independently (YG and YMC) screened the titles and abstracts of the records to determine if they met the inclusion criteria. Then, the same two authors retrieved the full text of all potentially eligible studies and assessed the eligibility of each study according to the inclusion criteria. Disagreements were resolved by discussion or by a third reviewer (JHT). When identified multiple studies from the same team or studies with samples from the same settings, we decided which study to include based on the study time frame and detailed data. For studies with overlapping data, we included studies with larger sample sizes.

### Data extraction and quality assessment

We used Microsoft Excel 2019 to construct a standard form to extract research data. The data abstracted included: (1) study characteristics (first author, year of publication, journal name, publication language, country of the first author, recruitment time frame, study design, study setting); (2) population characteristics (sex, age, sample size); (3) outcomes of interest (number of nervous system diseases patients, severe cases, non-severe cases, non-survivors, and survivors). The severe disease was defined as patients with acute respiratory distress syndrome (ARDS), needing mechanical ventilation, vital life support or intensive care unit admission [9–12]. We defined nervous system diseases according to the international classification of diseases -11 (ICD-11) [13, 14].

We used the Newcastle−Ottawa quality assessment scale (NOS) to assess the quality of the included studies [15]. Studies with more than 7 stars were regarded as high quality, 5–7 stars were regarded as moderate quality and lower than 5 stars were regarded as low quality. In our study, one reviewer (YG, YMC, ML or ZWS,) evaluated the quality of each study according to the scale and another (MLY and MMN) reviewed it. In the case of incongruity, the third researcher (JHT) was invited to discuss.

### Statistical analysis

We used Stata (13.0; Stata Corporation, College Station, Texas, USA Stata) to perform all meta-analyses. We conducted pairwise meta-analyses to compute the odds ratio (OR) with 95% confidence interval (95%CI) to estimate the association between nervous system diseases and COVID-19 severity or mortality. The meta-analyses used the inverse variance method with the random-effects model to estimate the average effect. We used the *I*^2^ statistic and Cochran's *Q* test to assess statistical heterogeneity. The *I*^2^ statistic results were interpreted as <25%, 26–50% and >50%, representing low, moderate and high heterogeneity, respectively [16].

Sensitivity analyses were applied by excluding studies published in Chinese to assess the stability of results. We further performed univariate meta-regression analyses to assess if the OR varied with study sample size. The funnel plot and Egger's test were used to detect publication bias for outcomes with studies no fewer than 10. The statistical level of significance was set at *P* < 0.05.

## Results

### Screening results

**Fig. 1.**
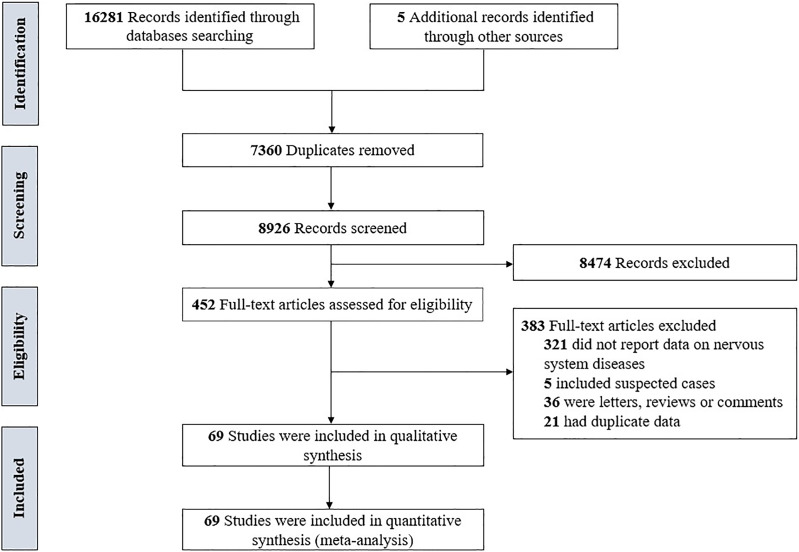
The flowchart of the screening process.

Totally, 16 286 records were identified through the literature search. After removing duplicates, 7360 records were excluded, and after reviewing the titles and abstracts, 8474 records were excluded. Through full-text evaluation of the remaining 452 records, 383 studies were further excluded, we finally included 69 studies [4, 17–84] in our meta-analyses. The flowchart of the screening process is presented in Fig. 1.

### General characteristics and quality of studies

All included studies were published online in 2020, incorporated patients between 11 December 2019 and 27 June 2020. In all, 68 studies [4, 17–83] were published in English and 1 study [84] published in Chinese. Out of which 54 studies [4, 18–24, 29–31, 34–39, 45–53, 55, 56, 59–84] were from China, 3 studies [17, 32, 54] were from the USA, 3 studies [25, 40, 44] were from Korea, 2 studies [26, 27] were from Italy and the remaining 7 studies [28, 33, 41–43, 57, 58] were from Austria, Iran, Israel, Saudi Arabia, Spain, Turkey, and UK. The sample size per study ranged from 27 to 1590 (total 17 879; 9686 males). Considering methodological quality in items of NOS scale, 23 studies [4, 17, 19, 21, 24, 27–29, 33, 34, 37, 46, 49, 55, 58, 61, 65, 67–69, 73, 75, 76] were rated as high quality (>7 stars) and 46 studies [18, 20, 22, 23, 25, 26, 30–32, 35, 36, 38–45, 47, 48, 50–54, 56, 57, 59, 60, 62–64, 66, 70–72, 74, 77–84] were rated as moderate quality (5−7 stars). The detailed characteristics and quality of the included studies are summarised in Table 1.

**Table 1.** Characteristics of included studies

Study	Year (Online)	Country	Publication language	Recruitment time frame	Sample	Age, years^a^	Sex		NOS
							Male	Female	
Argenziano MG [17]	2020	USA	English	2020.3.1–2020.4.15	1000	61.7 ± 17.5	596	404	8
Cao JL [18]	2020	China	English	2020.1.3–2020.2.1	102	54(37–67)	53	49	6
Chen T [19]	2020	China	English	2020.1.13–2020.2.12	274	62(44–70)	171	103	8
Chen TL [20]	2020	China	English	2020.1.1–2020.2.10	55	74(65–91)	34	21	6
Chen X [21]	2020	China	English	2020.2.3–2020.2.20	73	66(59–72.3)	42	31	8
Chen XH [22]	2020	China	English	2020.2.1–2020.2.19	48	64.6 ± 18.1	37	11	7
Cheng AY [23]	2020	China	English	2020.2.8–2020.3.11	305	65(52–71)	184	121	7
Cheng L [24]	2020	China	English	2020.1.3–2020.2.26	89	59.74 ± 16.42	49	40	8
Chon Y [25]	2020	Korea	English	2020.2.22–2020.4.3	281	61.5 ± 5.5	75	206	5
Colombi D [26]	2020	Italy	English	2020.2.17–2020.3.10	236	68(95%CI:66–70)	177	59	6
d'Arminio Monforte A [27]	2020	Italy	English	2020.2.24–2020.5.17	539	66(54–78)	347	192	9
Dupley L [28]	2020	UK	English	2020.3.1–2020.4.26	64	83 ± 9	29	35	8
Feng Y [29]	2020	China	English	2020.1.1–2020.2.15	476	53(40–64)	271	205	8
Fu J [30]	2020	China	English	2020.1.21–2020.3.4	35	47.94 ± 15	13	22	6
Gao S [31]	2020	China	English	2020.1.23–2020.2.29	210	71(67–77)	101	109	7
Gayam V [32]	2020	USA	English	2020.3.1–2020.4.9	408	67(56–76)	231	177	6
Götzinger F [33]	2020	Austria	English	2020.4.1–2020.4.24	582	5(0.5–12.0)	311	271	9
Guan WJ [34]	2020	China	English	2019.12.11–2020.1.31	1590	48.9 ± 16.3	904	686	8
Han MF [35]	2020	China	English	NR	154	42.4	86	68	7
Hu H [36]	2020	China	English	2020.2.7–2020.3.7	105	60.82 ± 16.32	62	43	7
Hu L [37]	2020	China	English	2020.1.8–2020.2.20	323	61(23–91)	166	157	8
Huang HF [38]	2020	China	English	2020.1.13–2020.3.10	64	47.8 ± 18.5	37	27	6
Huang Q [39]	2020	China	English	2020.1.17–2020.2.10	54	41(31–51)	28	26	7
Hwang JM [40]	2020	Korea	English	2020.2.1–2020.3.25	103	67.62 ± 15.32	52	51	6
Itelman E [41]	2020	Israel	English	2020.2–2020.4.10	162	52 ± 20	105	57	6
Javanian M [42]	2020	Iran	English	2020.2.25–2020.3.12	100	60.12 ± 13.87	51	49	6
Kutluhan MA [43]	2020	Turkey	English	2020.3.11–2020.5.10	96	58 ± 18.5	57	39	7
Lee JY [44]	2020	Korea	English	2020.2.21–2020.4.2	694	52.1 ± 18.29	212	482	7
Lei SQ [45]	2020	China	English	2020.1.2–2020.2.5	34	55(43–63)	14	20	7
Li M [46]	2020	China	English	2019.12.26–2020.2.25	245	54(37–64)	118	127	8
Li Q [47]	2020	China	English	2020.1.20–2020.2.29	325	51(36–64)	167	158	7
Li T [48]	2020	China	English	2020.2.1–2020.3.31	312	69.2 ± 7.3	187	125	6
Liu Q [49]	2020	China	English	2020.1.23–2020.2.29	84	51(37–59)	45	39	8
Liu SQ [50]	2020	China	English	2020.1.10–2020.3.15	625	44.44 ± 17.19	329	296	7
Lu L [51]	2020	China	English	2020.1.18–2020.2.18	304	44(33–59.25)	182	122	7
Luo XM [52]	2020	China	English	2020.1.30–2020.2.20	298	57(40–69)	150	148	7
Lyu PJ [53]	2020	China	English	2020.1.15–2020.2.14	51	54 ± 17	29	22	7
Maeda T [54]	2020	USA	English	2020.3.13–2020.3.31	224	63 ± 17	127	97	7
Pan L [55]	2020	China	English	2020.1.18–2020.2.28	103	52.91 ± 15.98	55	48	9
Qin C [56]	2020	China	English	2020.1.10–2020.2.12	452	58(47–67)	235	217	7
Romero-Sánchez CM [57]	2020	Spain	English	2020.3.1–2020.4.1	841	66.42 ± 14.96	473	368	6
Shabrawishi M [58]	2020	Saudi Arabia	English	2020.3.12–2020.3.31	150	46.1 ± 15.3	90	60	8
Wang CZ [59]	2020	China	English	2020.1.23–2020.2.13	45	39(16–62)	23	22	6
Wang D [60]	2020	China	English	2020.1.15–2020.2.28	143	58(39–67)	73	70	6
Wang DW(a) [4]	2020	China	English	2020.1.1–2020.1.28	138	56(42–68)	75	63	8
Wang DW(b) [61]	2020	China	English	−2020.2.10	107	51(36–65)	57	50	8
Wang F [62]	2020	China	English	2020.1–2020.3	108	Survivors 70.9 ± 10.6/non-survivors 71.1±10.1	72	36	7
Wang L [63]	2020	China	English	2020.1.1–2020.2.6	339	69(65–76)	168	171	5
Wang LW [64]	2020	China	English	2020.1.14–2020.2.13	116	54(38–69)	67	49	6
Wang WL [65]	2020	China	English	2020.2.10–2020.3.27	123	68(56.5–78)	60	63	8
Wang YF [66]	2020	China	English	2020.1-2020.2.10	110	NR	48	62	7
Wang YP [67]	2020	China	English	2020.1.20–2020.2.10	275	49(34–62)	128	147	8
Wei YP [68]	2020	China	English	2020.1.27–2020.3.11	276	51(41–58)	155	121	9
Wu GY [69]	2020	China	English	2019.12.23–2020.2.13	299	50(35.5–63)	137	162	8
Wu J [70]	2020	China	English	2020.1.20–2020.2.20	280	43.12 ± 19	151	129	7
Wu SR [71]	2020	China	English	2020.1.27–2020.2.26	270	62(50–69)	139	131	6
Xie JF [72]	2020	China	English	2020.1.1–2020.2.29	733	65(56–73)	477	256	7
Yan XQ [73]	2020	China	English	2020.1.21–2020.6.27	218	42.9(32–52.3)	122	96	9
Yan YL [74]	2020	China	English	2020.1.10–2020.2.24	193	64(49–73)	114	79	7
Yang QX [75]	2020	China	English	2020.1.28–2020.2.12	136	56(44–64)	66	70	8
Yang XB [76]	2020	China	English	2019.12.24–2020.1.26	52	59.7 ± 13.3	35	17	8
Yuan ML [77]	2020	China	English	2020.1.1–2020.1.25	27	60(47–69)	12	15	6
Zhang GQ [78]	2020	China	English	2020.1.2–2020.2.10	221	55(39–66.5)	108	113	7
Zhang HM [79]	2020	China	English	2020.1.28–2020.2.24	88	55(22–89)	45	43	7
Zhang JJ [80]	2020	China	English	2020.1.16–2020.2.3	140	57(25–87)	71	69	7
Zhang L [81]	2020	China	English	2020.1.20–2020.2.29	409	65(56–71)	234	175	6
Zhao Y [82]	2020	China	English	2020.1.13- 2020.3.4	539	58(43–69)	255	284	6
Zheng F [83]	2020	China	English	2020.1.17–2020.2.7	161	45(33.5–57)	80	81	6
Zou WB [84]	2020	China	Chinese	2020.2.1–2020.2.29	63	Severe 52 ± 16/non-severe 43±16	32	31	6

a Age data presented as median (IQR) or mean ± s.d.. NR, not reported.

### Association between nervous system diseases and the severity and mortality of COVID-19

**Fig. 2.**
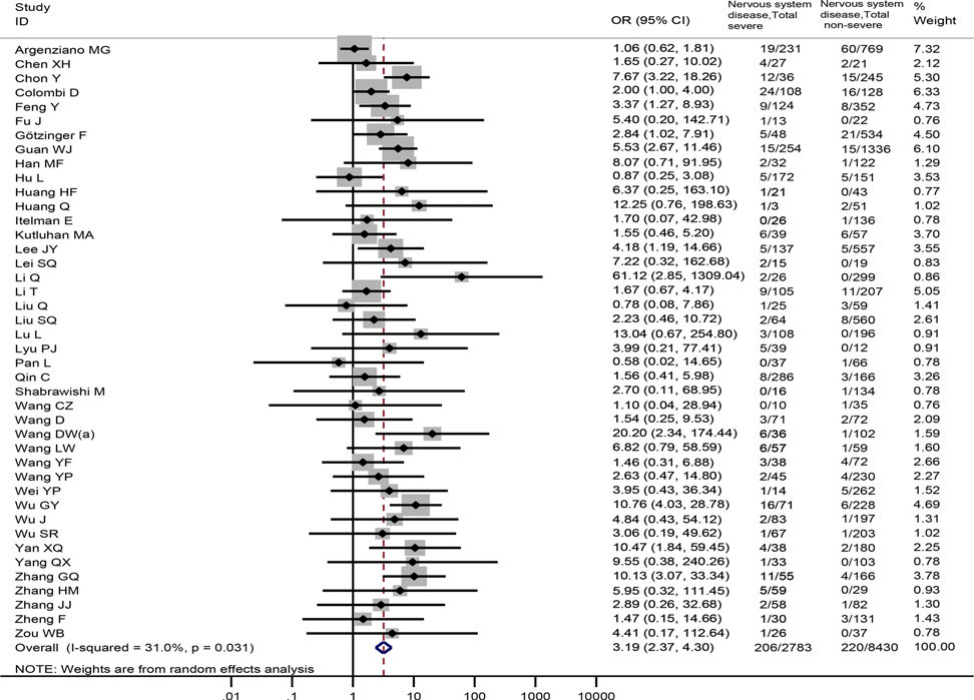
Association between nervous system diseases and the severity of COVID-19.

In all, 42 studies [4, 17, 22, 25, 26, 29, 30, 33–35, 37–39, 41, 43–45, 47–51, 53, 55, 56, 58–60, 64, 66–71, 73, 75, 78–80, 83, 84] totaling 11 213 patients reported prevalence of nervous system diseases among COVID-19 patients with the severe and non-severe disease. The meta-analysis demonstrated that nervous system diseases were associated with COVID-19 severity (OR = 3.19, 95%CI: 2.37 to 4.30, *P* < 0.001; *I*^2^ = 31.0%) (Fig. 2). We observed a significant association (OR = 3.19, 95%CI: 2.36 to 4.32, *P* < 0.001) between nervous system diseases and COVID-19 severity after excluding a Chinese study [84] (Appendix Fig. 1).

**Fig. 3.**
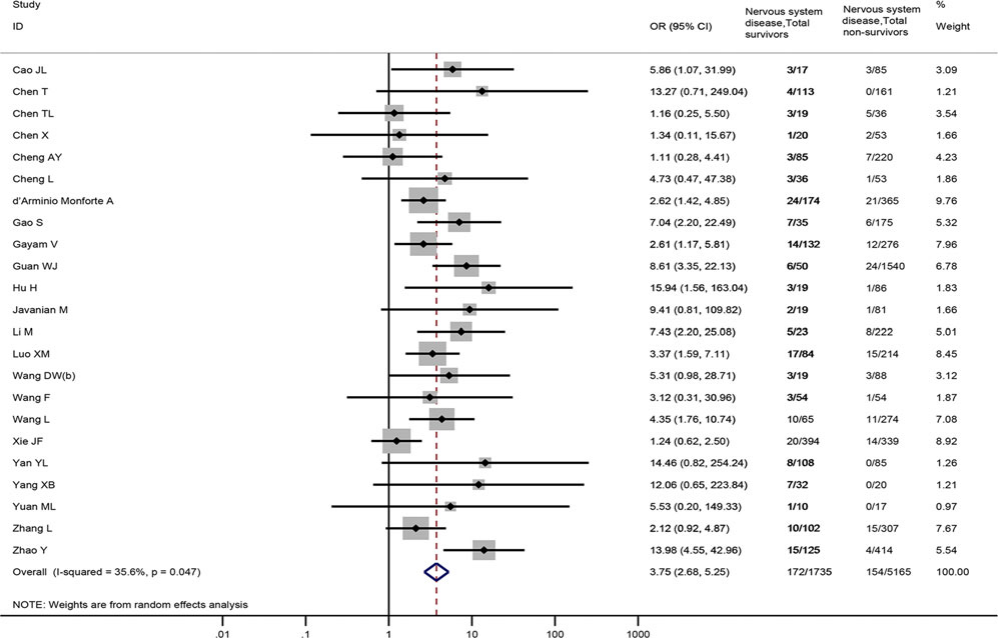
Association between nervous system diseases and the mortality of COVID-19.

Overall, 23 studies [18–21, 23, 24, 27, 31, 32, 34, 36, 42, 46, 52, 61–63, 72, 74, 76, 77, 81, 82], involving 6900 patients provided nervous system diseases data between non-survivors and survivors. The result revealed that nervous system diseases were associated with a significantly enhanced risk of death (OR = 3.75, 95%CI: 2.68 to 5.25, *P* < 0.001; *I*^2^ = 35.6%) (Fig. 3).

### Association between cerebrovascular disease and the severity and mortality of COVID-19

**Fig. 4.**
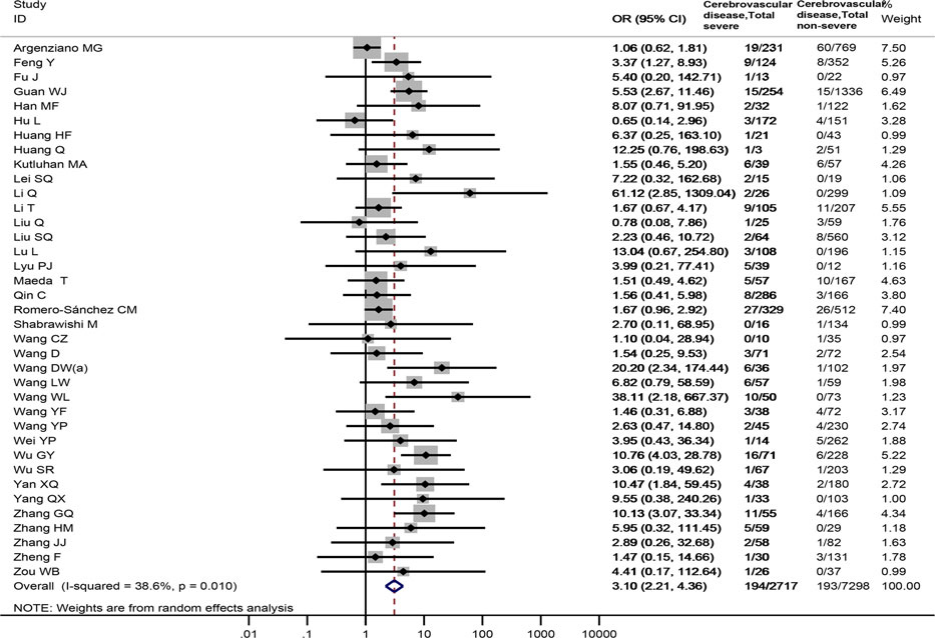
Association between cerebrovascular disease and the severity of COVID-19.

Thirty-seven studies [4, 17, 29, 30, 34, 35, 37–39, 43, 45, 47–51, 53, 54, 56–60, 64–69, 71, 73, 75, 78–80, 83, 84], totaling 10 015 samples, reported the prevalence of cerebrovascular disease between severe and non-severe COVID-19 patients. Cerebrovascular disease was observed to be associated with a significantly enhanced risk of severe COVID-19 disease (OR = 3.10, 95%CI: 2.21 to 4.36, *P* < 0.001; *I*^2^ = 38.6%), Fig. 4. Sensitivity analysis by excluding a Chinese study [84] showed similar results (OR = 3.10, 95%CI: 2.19 to 4.39), Appendix Fig. 2.

**Fig. 5.**
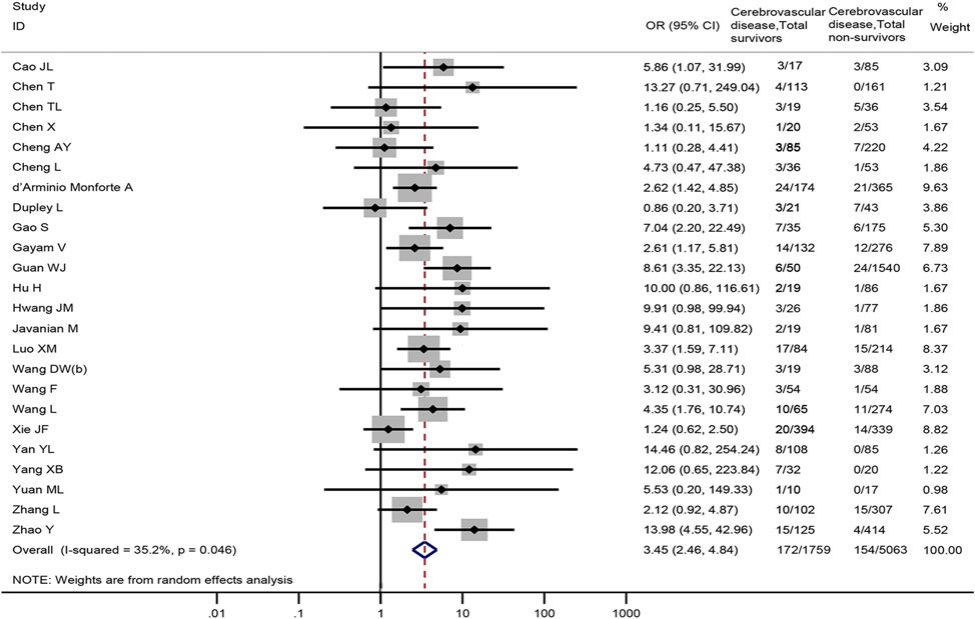
Association between cerebrovascular disease and the mortality of COVID-19.

Twenty-four studies [18–21, 23, 24, 27, 28, 31, 32, 34, 36, 40, 42, 52, 61–63, 72, 74, 76, 77, 81, 82], including 6822 patients, reported cerebrovascular disease data between non-survivors and survivors. The meta-analysis demonstrated that cerebrovascular disease was associated with death in COVID-19 patients (OR = 3.45, 95% CI: 2.46 to 4.84, *P* < 0.001; *I*^2^ = 35.2%) (Fig. 5).

### Association between stroke, epilepsy, dementia and the severity and mortality of COVID-19

**Fig. 6.**
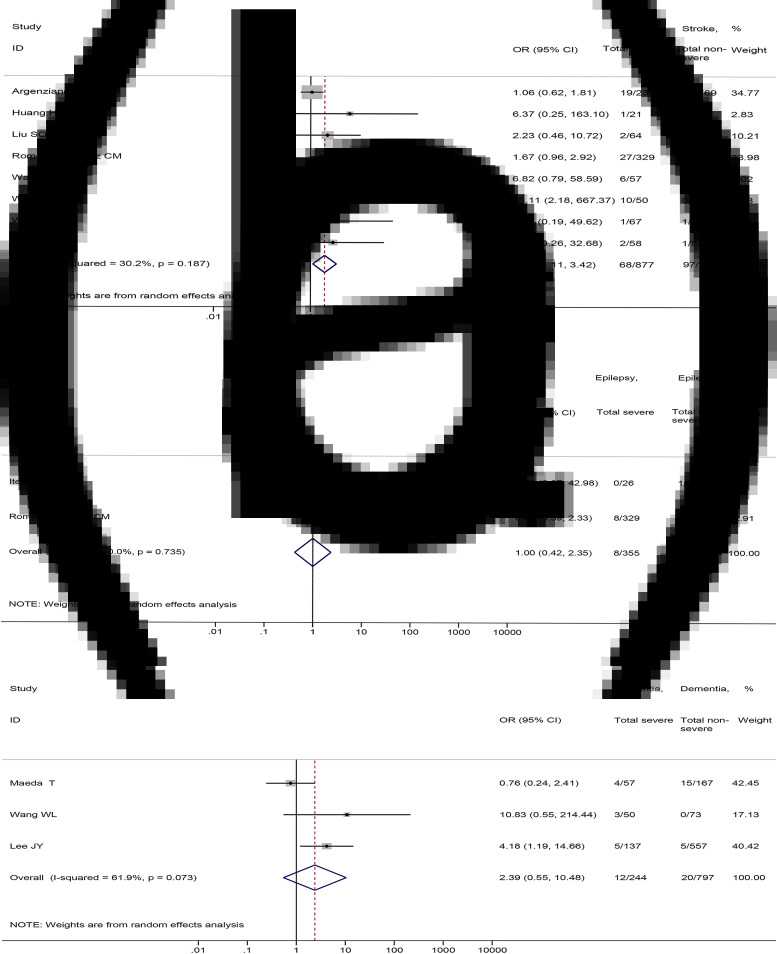
Association between (a) stroke, (b) epilepsy, (c) dementia and the severity of COVID-19.

As for specific nervous system diseases, our meta-analysis showed that stroke was associated with severe COVID-19 disease (8 studies [17, 38, 50, 57, 64, 65, 71, 80], 3178 patients; OR = 1.95, 95%CI: 1.11 to 3.42, *P* = 0.020; *I*^2^ = 30.2%) (Fig. 6A). There were no significant differences in the prevalence of epilepsy (2 studies [41, 57], 1003 patients; OR = 1.00, 95%CI: 0.42 to 2.35, *P* = 0.994; *I*^2^ = 0.0%) and dementia (3 studies [44, 54, 65], 1041 patients; OR = 2.39, 95%CI: 0.55 to 10.48, *P* = 0.247; *I*^2^ = 61.9%) between severe and non-severe patients (Fig. 6B and C).

**Fig. 7.**
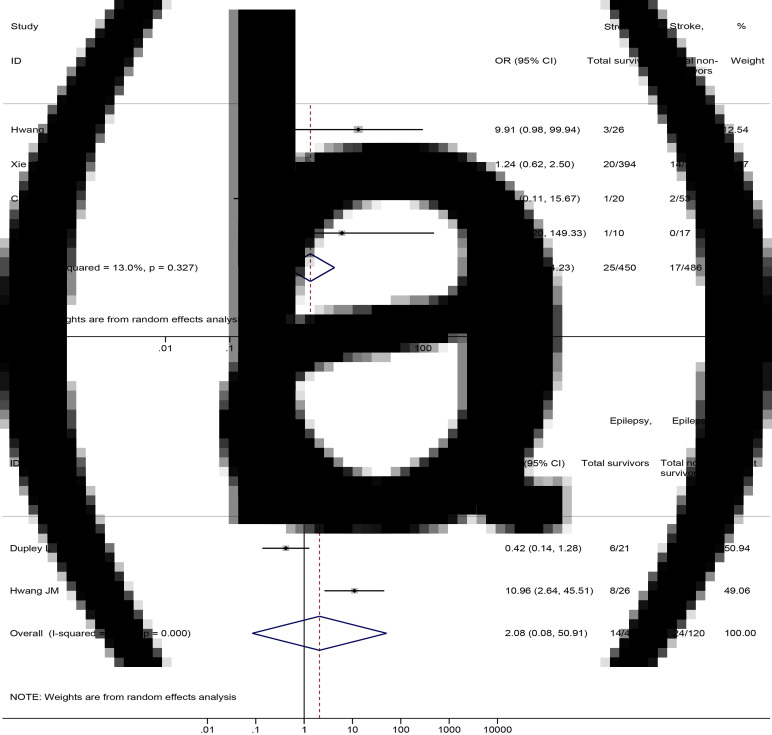
Association between (a) stroke and (b) epilepsy and the mortality of COVID-19.

No significant differences were found in the prevalence of stroke (4 studies [21, 40, 72, 77], 936 patients; OR = 1.79, 95%CI: 0.76 to 4.23, *P* = 0.185; *I*^2^ = 13.0%) and epilepsy (2 studies [28, 40], 167 patients; OR = 2.08, 95%CI: 0.08 to 50.91, *P* = 0.654; *I*^2^ = 92.0%) between non-survival and survival patients (Fig. 7).

### Meta-regression analyses

Univariate meta-regression analyses revealed that the sample size of individual study was not the source of heterogeneity or the factor affecting the association between nervous system diseases and COVID-19 severity or mortality (Appendix Figs 3 and 4) and the association between cerebrovascular disease and COVID-19 severity or mortality (Appendix Figs. 5 and 6).

### Publication bias

The funnel plot and Egger's test revealed that there was no statistically significant publication bias of nervous system diseases associated with severity (*P* = 0.090) (Appendix Fig. 7) and mortality of COVID-19 (*P* = 0.061) (Appendix Fig. 8). We found that there was a possibility of publication bias for the association between cerebrovascular disease and COVID-19 severity (*P* = 0.011) (Appendix Fig. 9). There was no statistically significant publication bias for the association between cerebrovascular disease and COVID-19 mortality (*P* = 0.100) (Appendix Fig. 10).

## Discussion

### Principal findings

This study included 69 studies and systematically assessed the association between nervous system diseases and the severity and mortality of patients with COVID-19. Specifically, we also conducted meta-analyses to explore the association between cerebrovascular disease and severity or mortality of patients with COVID-19, as well as the association between stroke, epilepsy, dementia and COVID-19 severity and mortality. Our meta-analyses revealed that nervous system diseases were associated with severity and mortality of patients with COVID-19. Cerebrovascular disease was associated with severity and mortality of patients with COVID-19. Severe COVID-19 patients were more likely to have a stroke compared with non-severe patients. There were no significant associations between epilepsy and dementia and COVID-19 severity or mortality. Sensitivity analyses suggested that the results did not change substantially after excluding studies published in Chinese.

### Comparison with other studies

A previous meta-analysis, including three studies with a total sample size of 1299, demonstrated that a significant relationship between patients with severe COVID-19 and cerebrovascular disease (OR = 3.89, 95% CI: 1.64 to 9.22, *P* = 0.002) [85]. Another meta-analysis, including seven studies involving 2585 patients, showed that cerebrovascular disease was significantly associated with severe COVID-19 disease (RR = 1.88, 95% CI: 1.00 to 3.51, *P* = 0.05) and five studies involving 936 patients revealed that cerebrovascular disease was associated with COVID-19 mortality (RR = 2.38, 95%CI: 1.92 to 2.96, *P* < 0.001) [5]. Compared with these two studies, our study reached similar conclusions. However, it has distinct advantages and our results are more comprehensive. Our study meta-analysed 37 studies involving a total of 10 015 COVID-19 patients between cerebrovascular disease and COVID-19 severity, at the same time, 24 studies with a total sample size of 6822 between cerebrovascular disease and COVID-19 mortality. Therefore, our meta-analysis has the advantage of expanding the sample size and including more research studies. To the best of our knowledge, the two previous meta-analyses that included studies completely came from China. In this study, we included 15 studies from the USA, Korea, Italy, UK, Austria, Iran, Israel, Saudi Arabia, Spain and Turkey, which expanded our research scope. Another difference between our study and previous meta-analyses is that we also analysed the relationship between detailed nervous system diseases including stroke, epilepsy, dementia and the severity and mortality of patients with COVID-19. Furthermore, we also performed sensitivity analyses and meta-regression analyses and investigated the publication bias, and these analyses indicated that results of our study were stable. Therefore, the results of our study are more systematic and comprehensive.

### Implications for research and practice

Previous studies have reported that SARS and Middle East respiratory syndrome (MERS) patients with nervous system diseases are at a higher risk of poor outcomes [86, 87]. Our study revealed that nervous system diseases were associated with severity and mortality of patients with COVID-19. Previous studies have shown that SARS-CoV-1 can invade the nerves and cause direct central nervous system infection [88, 89], which may also be one of the pathogenic pathways of SARS-CoV-2. Furthermore, the SARS-CoV-2 virus may enter the cerebral circulation, and the interaction between the viral spike proteins and the ACE2 receptors expressed in the brain capillary endothelium may destroy the blood−brain barrier [90, 91]. SARS-CoV-2 can infect cardiomyocytes through ACE2 receptors and cause vascular damage and inflammation, making thrombus easy to form and increasing the risk of stroke [92, 93]. COVID-19 could also cause viral encephalitis and haemorrhagic necrosis in the mesial temporal lobes and thalamus [93]. These may be the potential mechanisms for the poor prognosis of COVID-19 patients with nervous system diseases. However, the exact mechanism of increased severity of COVID-19 in patients with nervous system diseases remains unclear, which requires further research to clarify.

Our meta-analyses found that cerebrovascular disease was associated with severity and mortality of patients with COVID-19. These findings highlight the need for neurologists to be vigilant to the high risk of serious illness and death associated with COVID-19 infection in patients with nervous system diseases. A systematic review showed that an increasing number of reports of COVID-19 patients with neurological disorders have added emergent experimental models with neuro-invasion, which is a reasonable concern because SARS-CoV-2 is a new neuropathogen [94]. However, at present, there is a lack of treatment strategies for COVID-19 patients with nervous system diseases. Therefore, protecting patients with nervous system diseases from COVID-19 is a problem worthy of our attention. To the best of our knowledge, there is currently no recommendation regarding the treatment strategies for nervous system diseases patients with COVID-19. The results of our meta-analysis also provide the latest references for the development of new guidelines. There is an urgent need for high-quality evidence-based guidelines to clarify the protective measures for patients with nervous system diseases, as well as care and treatment strategies for nervous system disease patients with COVID-19.

### Strengths and limitations

Despite comprehensive analyses, our meta-analysis has many limitations. First, we found that some patients of included studies were still hospitalised at the end of the study and no studies reported the specific time period of nervous system diseases. Second, since we included cohort studies and case−control studies, there might be confounding factors that influence the relationship between nervous system diseases and COVID-19 severity as well as mortality. Third, there was much variation in eligibility for SARS-CoV-2 testing between studies or over time within studies. Fourth, we conducted meta-regression analysis and sensitivity analysis to explore the sources of heterogeneity, but the selected factors were not the sources of heterogeneity and the results of some meta-analyses may be affected by the high heterogeneity. Finally, the total number of patients with nervous system diseases included in analyses is relatively small even in this comprehensive literature review, resulting in some wide confidence intervals. As described above, these limitations showed that caution is required before drawing any firm conclusions in the absence of high-quality, comprehensive evidence.

## Conclusions

Nervous system diseases were associated with severity and mortality of patients with COVID-19. Among them, cerebrovascular disease was associated with a high risk of severity and mortality of patients with COVID-19. However, due to the limitations of this study, more high-quality, large sample, multicentre trials are needed to provide robust evidence to support clinical practice.

## Data Availability

All datasets generated for this study are included in the manuscript.

## Supplementary material

For supplementary material accompanying this paper visit http://dx.doi.org/10.1017/S0950268821000376. click here to view supplementary material
