# Supplementary material for: Nervous system diseases are associated with the severity and mortality of patients with COVID-19: a systematic review and meta-analysis
Source: Epidemiol Infect. 2021 Feb 15;149:e66. doi: 10.1017/S0950268821000376 (PMC7985867; doi:10.1017/S0950268821000376)

**Appendix Word 1.** Search strategy of PubMed.

#1 "COVID-19"[Supplementary Concept] OR "severe acute respiratory syndrome

coronavirus 2"[Supplementary Concept]

#2 COVID-19[Title/Abstract] OR COVID 19[Title/Abstract] OR 2019 novel coronavirus[Title/Abstract] OR 2019-nCov[Title/Abstract] OR coronavirus disease 2019[Title/Abstract] OR coronavirus disease-19[Title/Abstract] OR new coronavirus[Title/Abstract] OR novel corona virus[Title/Abstract] OR nCoV-2019[Title/Abstract] OR novel coronavirus pneumonia[Title/Abstract] OR "severe acute respiratory syndrome coronavirus 2"[Title/Abstract] OR SARS-CoV-2[Title/Abstract]

#3 #1 OR #2

#4 clinical characteristics[Title/Abstract] OR clinical characteristic[Title/Abstract] OR clinical feature[Title/Abstract] OR clinical features[Title/Abstract] OR risk factors[Title/Abstract] OR risk factor[Title/Abstract] OR prognosis[Title/Abstract] OR comorbidit*[Title/Abstract] OR cerebrovascular disease*[Title/Abstract] OR nervous system disease*[Title/Abstract] OR brain[Title/Abstract] OR neurologic* [Title/Abstract] OR stroke[Title/Abstract] OR cerebral infarction[Title/Abstract] OR epilepsy[Title/Abstract] OR dementia[Title/Abstract] OR severity[Title/Abstract] OR mortality[Title/Abstract]

#5 #3 AND #4

**Appendix Figure** **1.** Sensitivity analysis by excluding Chinese studies for the association between nervous system diseases and COVID-19 severity


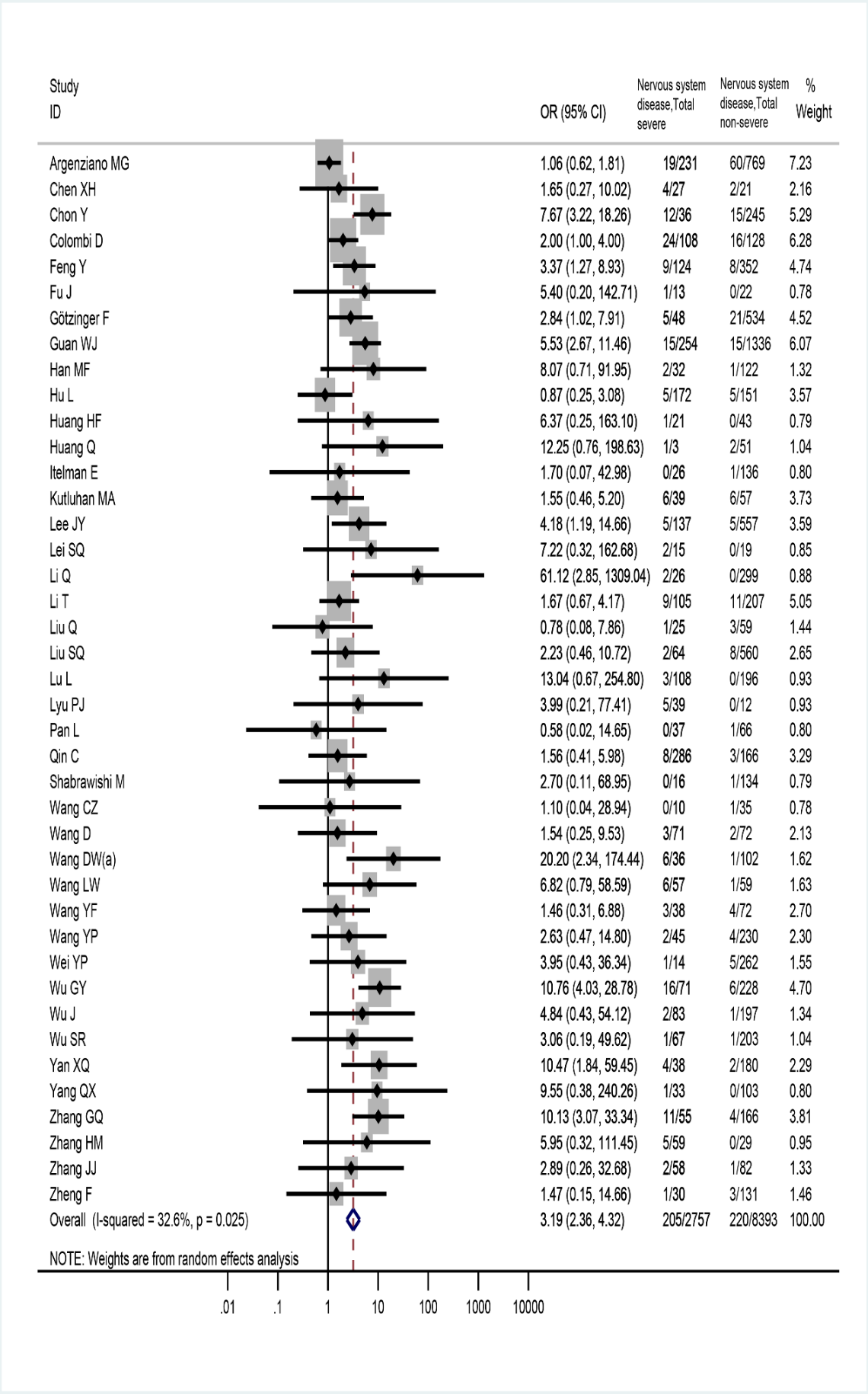


**Appendix Figure 2.** Sensitivity analysis by excluding Chinese studies for the association between cerebrovascular diseases and COVID-19 severity


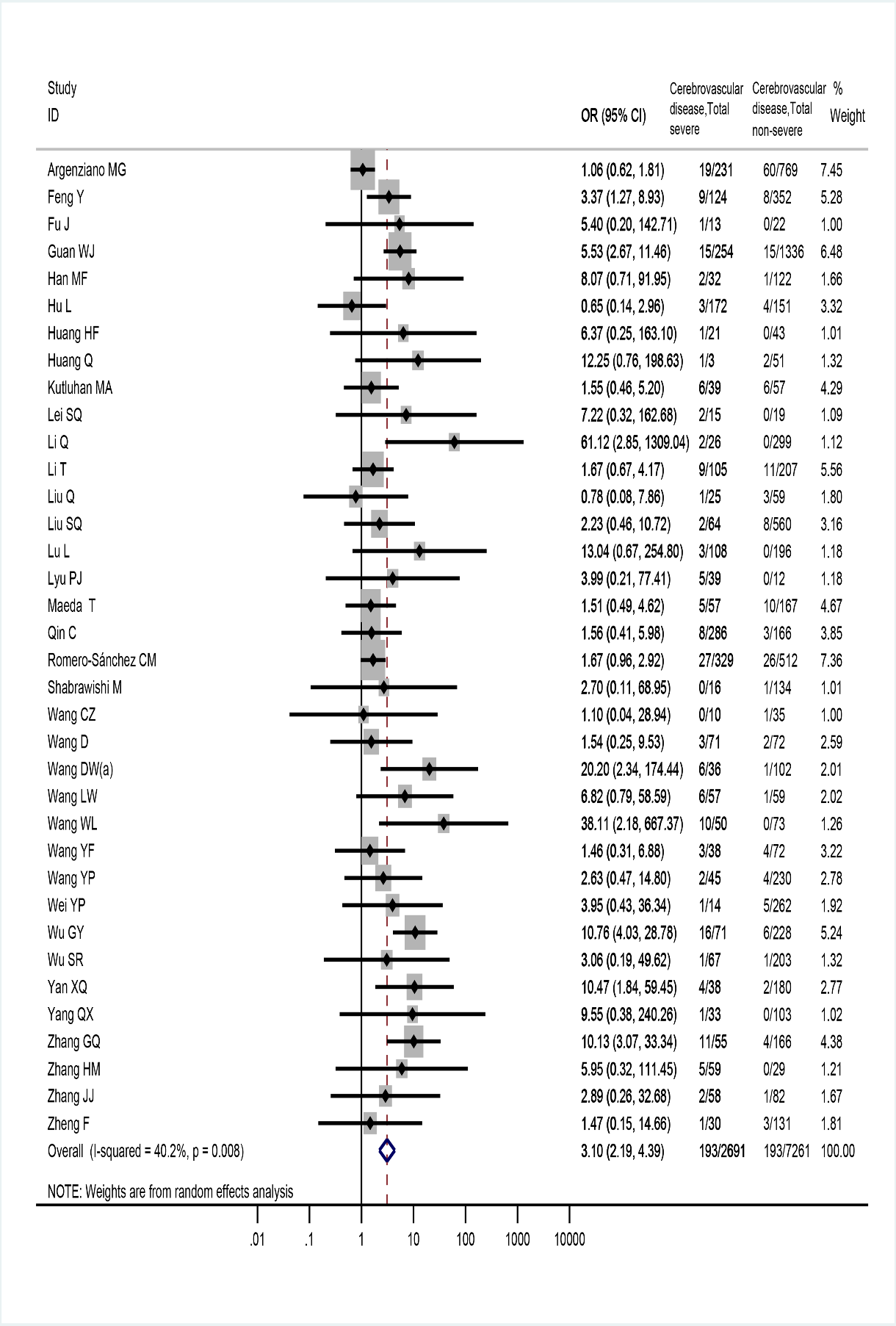


**Appendix Figure 3.** Univariate meta-regression analysis on the sample size of each study for the association between nervous system diseases and COVID-19 severity


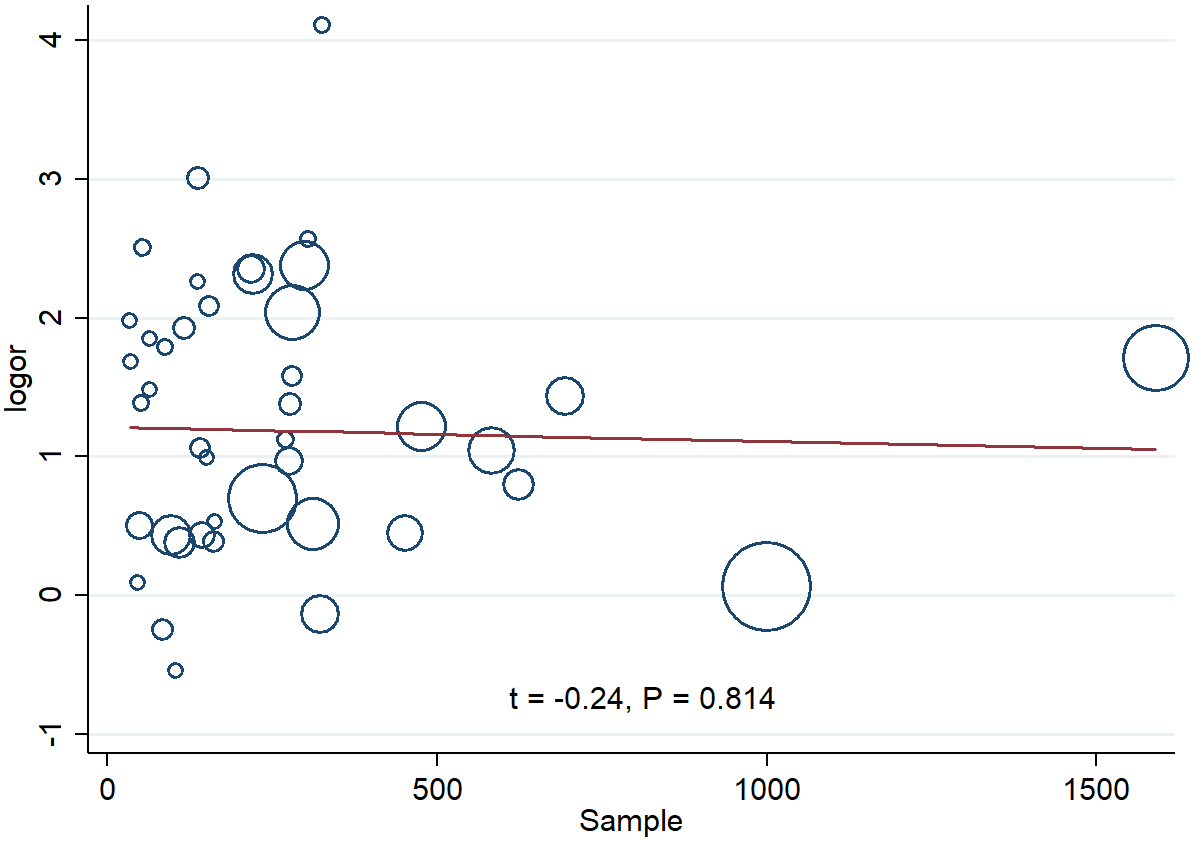


**Appendix Figure 4.** Univariate meta-regression analysis on the sample size of each study for the association between nervous system diseases and COVID-19 mortality


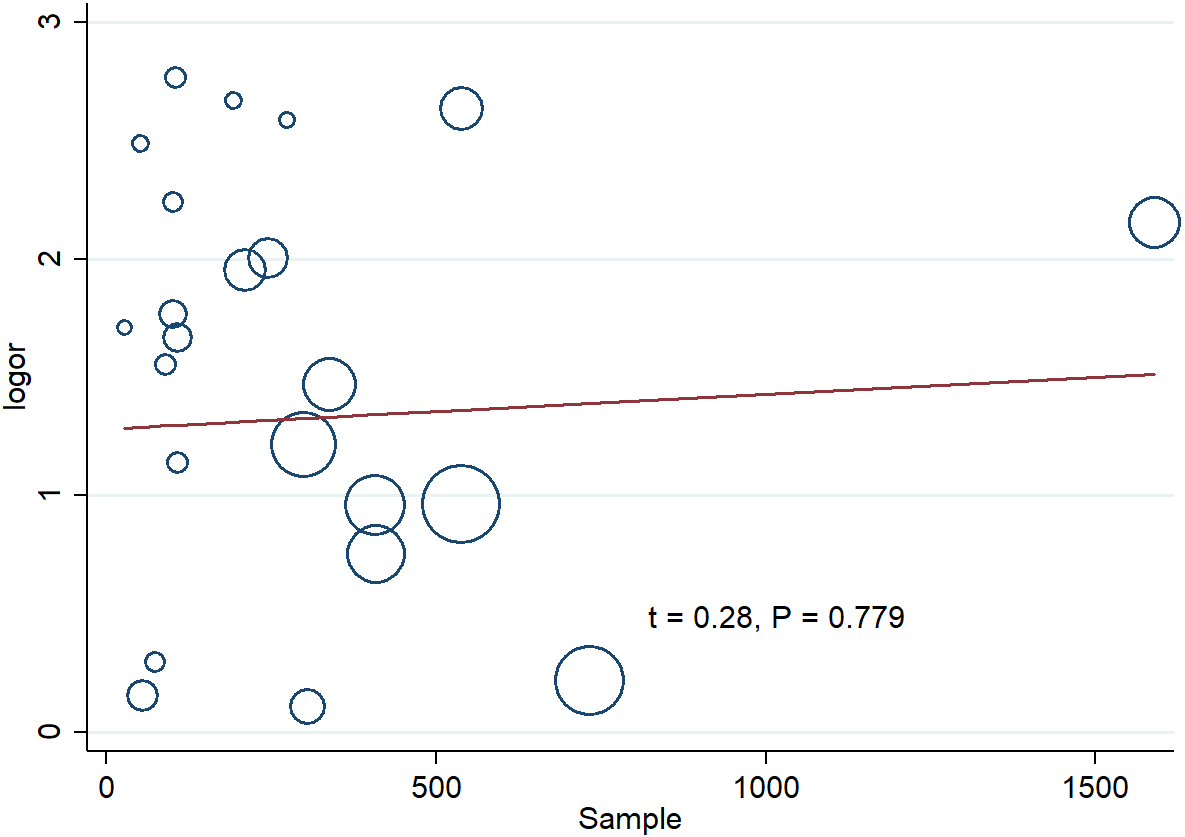


**Appendix Figure 5.** Univariate meta-regression analysis on the sample size of each study for the association between cerebrovascular diseases and COVID-19 severity


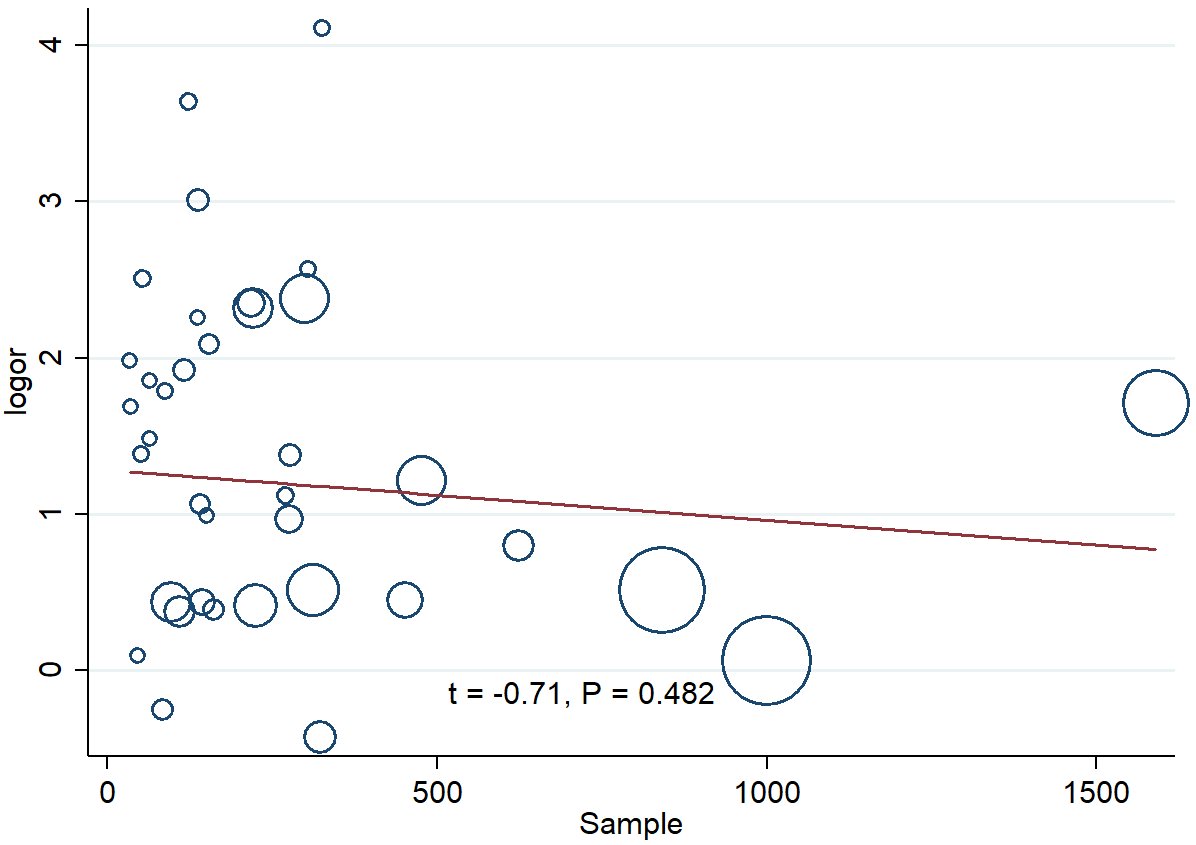


**Appendix Figure 6.** Univariate meta-regression analysis on the sample size of each study for the association between cerebrovascular diseases and COVID-19 mortality


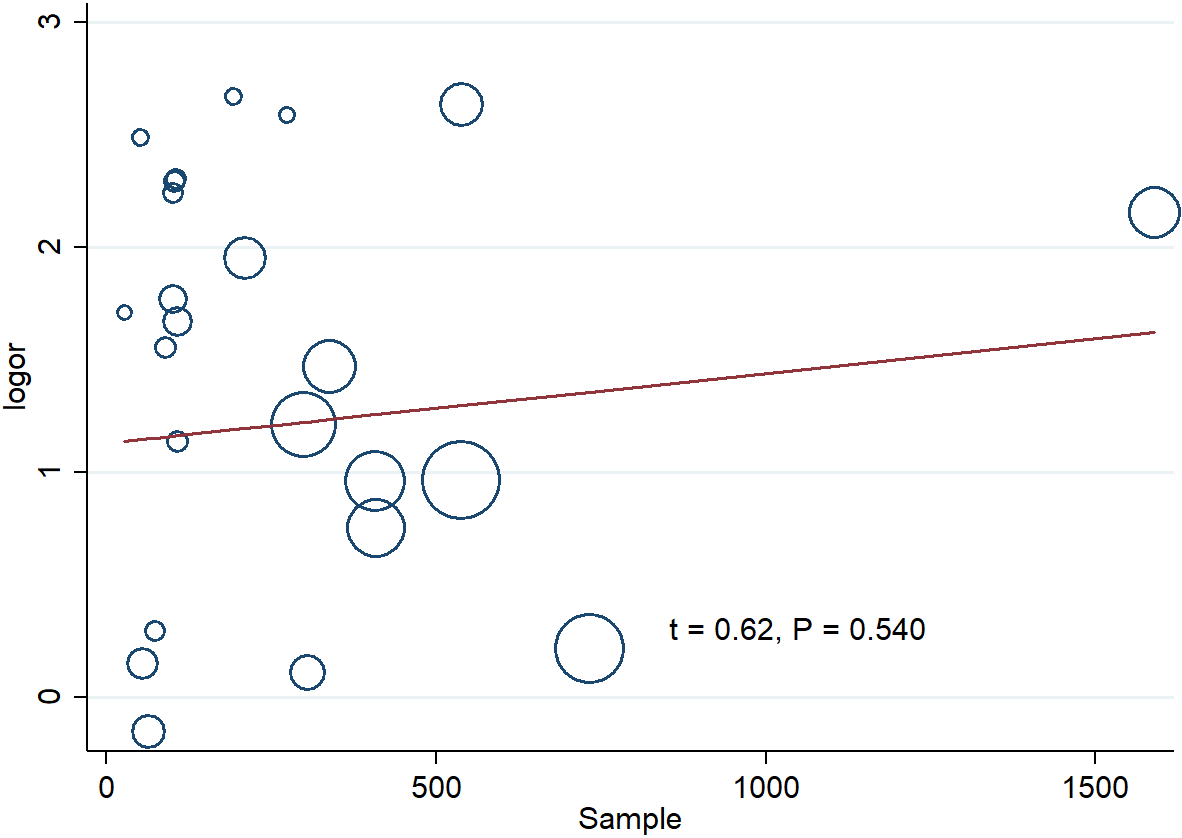


**Appendix Figure 7.** Funnel plot for the association between nervous system diseases and COVID-19 severity


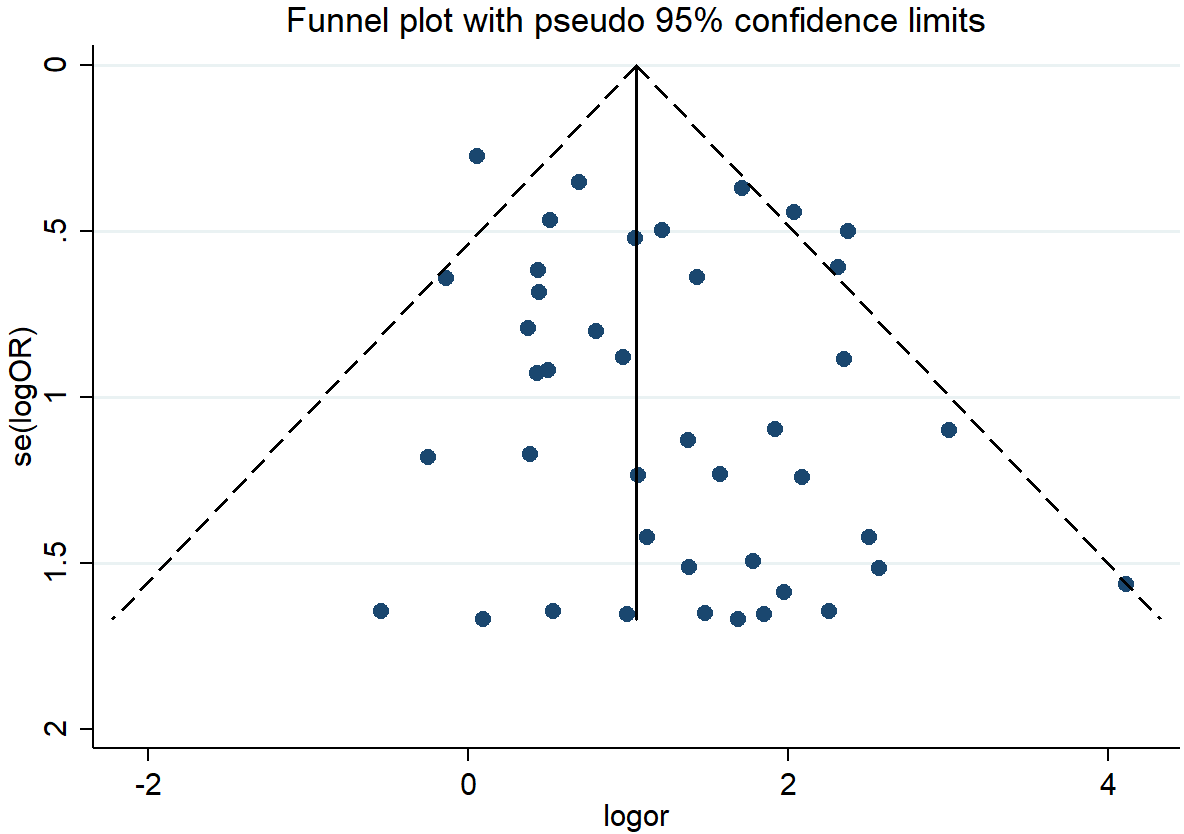


**Appendix Figure 8.** Funnel plot for the association between nervous system diseases and COVID-19 mortality


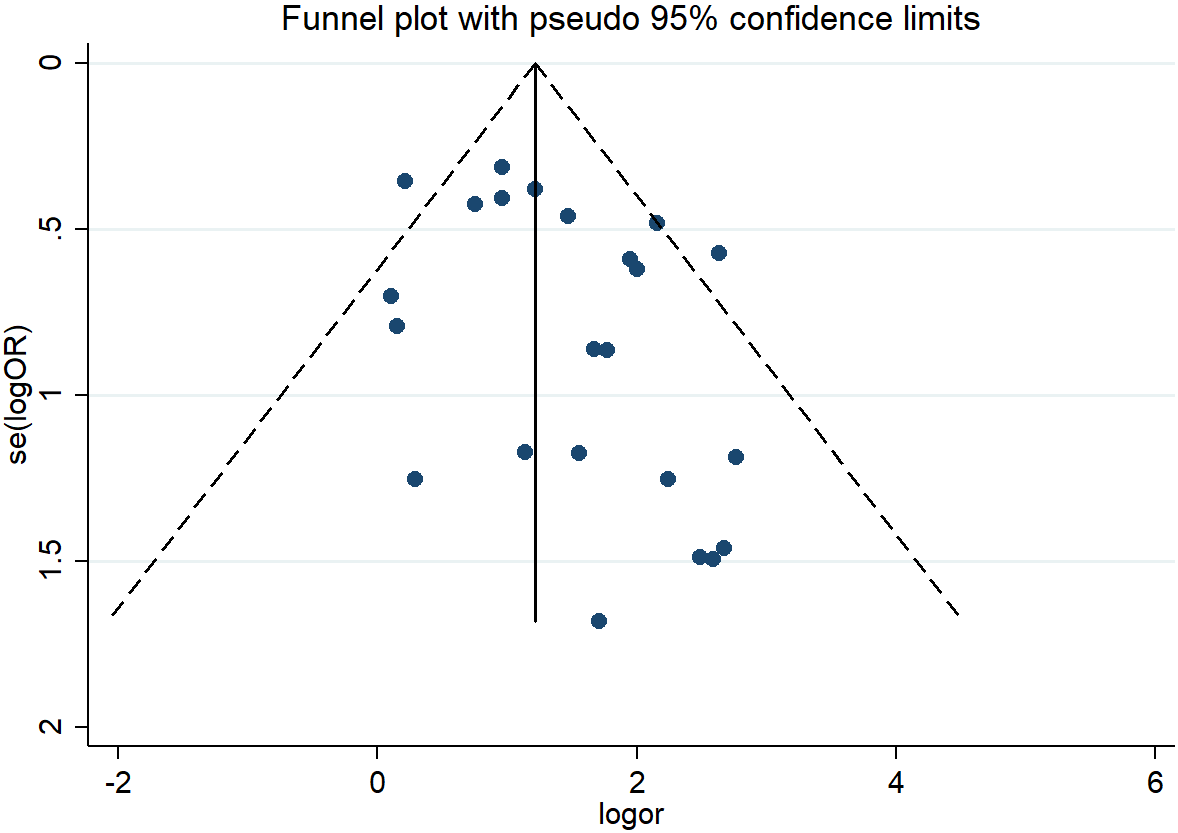


**Appendix Figure 9.** Funnel plot for the association between cerebrovascular disease and COVID-19 severity


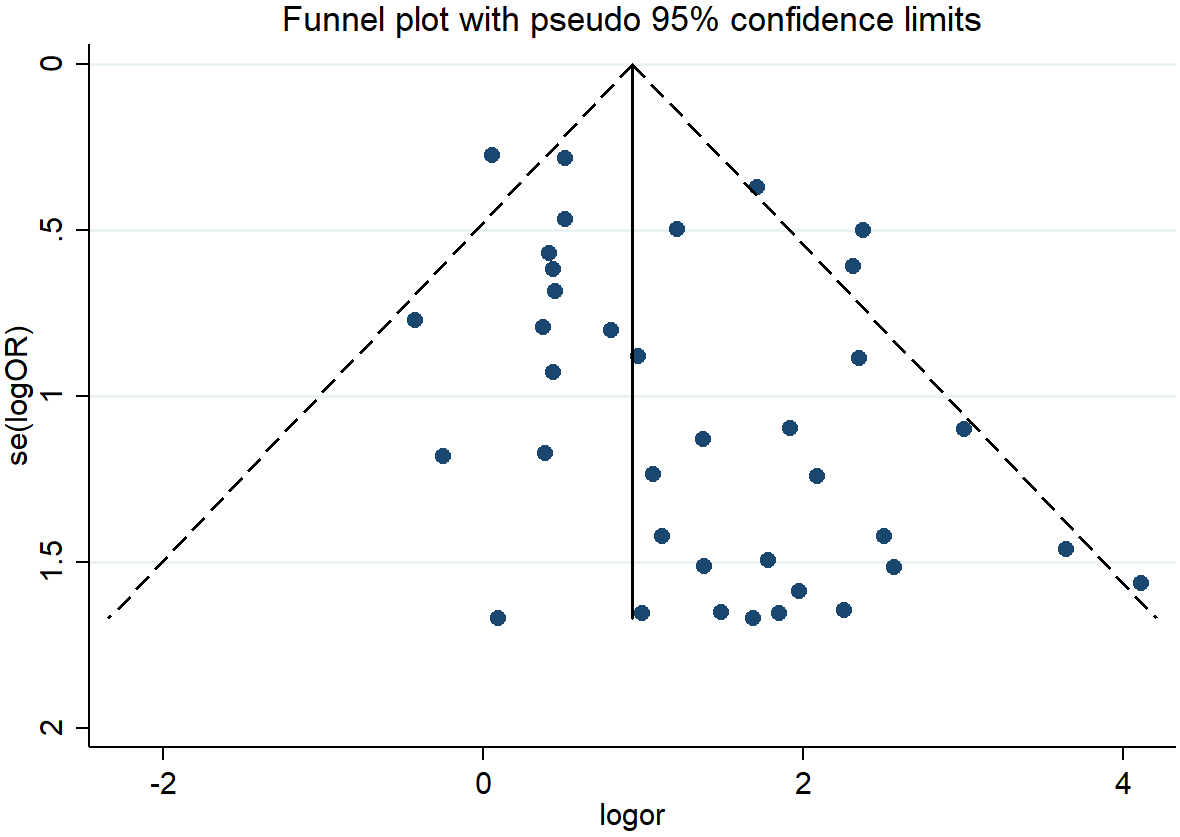


**Appendix Figure 10.** Funnel plot for the association between cerebrovascular diseases and COVID-19 mortality


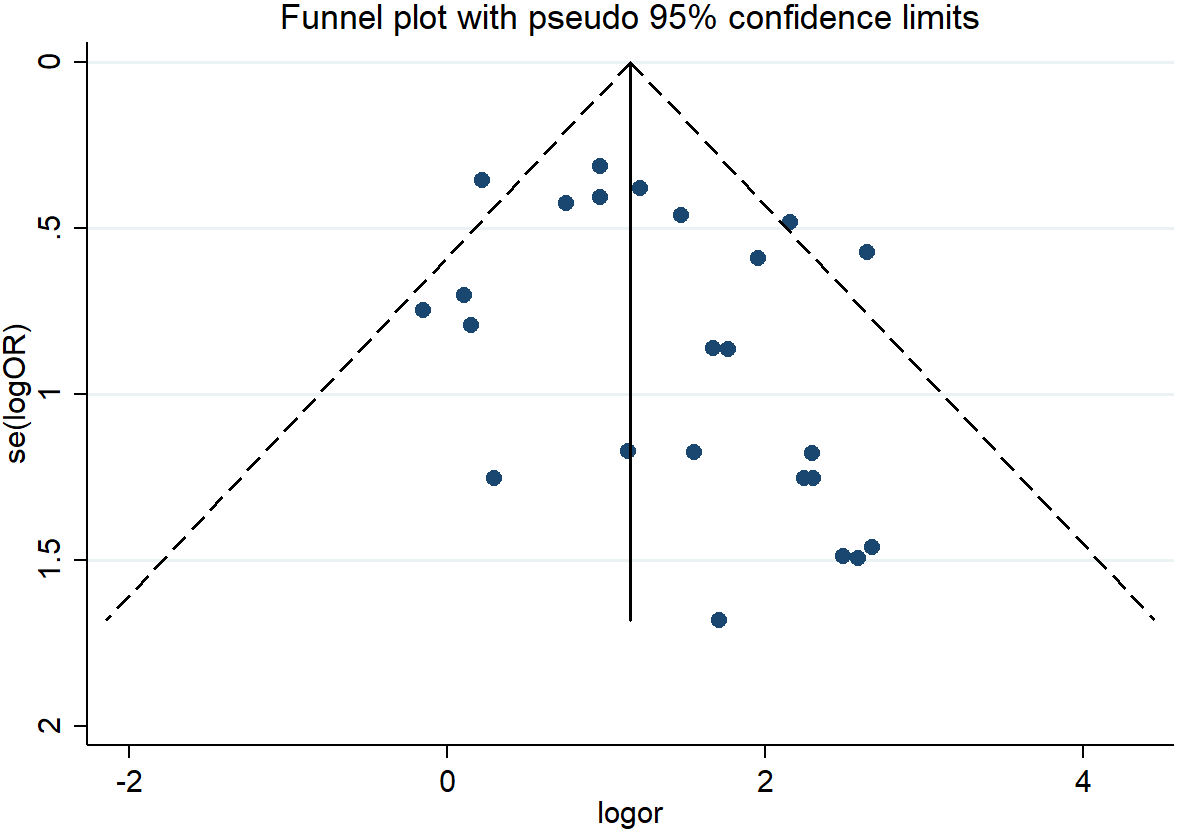

Supplement: Supplementary file 1 [file S0950268821000376sup001.docx]
